# Photopolymer Flexographic Printing Plate Mold for PDMS Microfluidic Manufacture

**DOI:** 10.3390/polym17131723

**Published:** 2025-06-20

**Authors:** Ana Belén Peñaherrera-Pazmiño, Gustavo Iván Rosero, Maximiliano Pérez, Betiana Lerner

**Affiliations:** 1 Centro de Investigación Biomédica (CENBIO), Facultad de Ciencias de la Salud Eugenio Espejo, Universidad UTE, Quito 170527, Ecuador; 2 Special Coatings and Nanostructures Engineering (IREN), National Technological University, Buenos Aires 1706, Argentina; gustavorosero@gmail.com (G.I.R.); maxperez@fiu.edu (M.P.); 3 Collaborative Research Institute Intelligent Oncology (CRIION), Hermann-Herder-Straße 4, 79104 Freiburg im Breisgau, Germany; 4 Department of Electrical and Computer Engineering, Florida International University, Miami, FL 33174, USA

**Keywords:** flexography, microfluidics, sustainable development goal 3, sustainable development goal 10, biomedicine, cell culture

## Abstract

Flexographic printing, traditionally used in the packaging industry, has emerged as a promising technology for microfluidic device fabrication due to enabling high resolution and being commercially available at a low cost compared to conventional techniques. This review explores the adaptation of a photopolymer flexographic printing plate mold (FMold) for microfluidics, examining its advantages, challenges, and applications. It offers a state-of-the-art view of the application of FMold for microfluidic systems, which offers a unique opportunity in terms of cost-effectiveness, scalability, and rapid prototyping. Applications are diverse: FMold has enabled the fabrication of microfluidic devices used in enhanced oil recovery to prepare rock-on-a-chip models, droplet generation and storage, suspension cell culture, monoclonal antibody production, complex cell differentiation pattern creation, phage screening, drug screening, cell detection, and cancer stem cell culture. Since its first appearance in 2018, FMold has been utilized in 50 publications in different laboratories around the world. Key advancements, current research trends, and future prospects are discussed to provide a comprehensive overview of this evolving tool.

## 1. Introduction

### Flexographic Printing: Fundamentals and Adaptation

**Figure 1:**
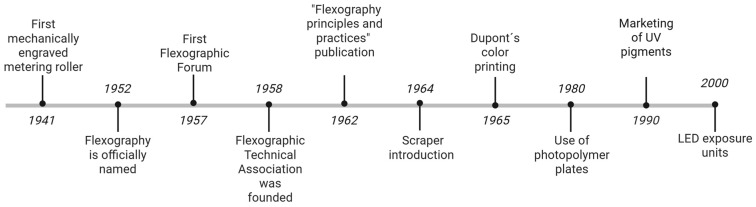
Flexography timeline. Image based on [2]. Created in BioRender.com.

Flexography has its roots in the late 19th century “aniline printing” method, utilizing rotary presses with rubber plates and aniline-based inks [1]. In 1941, Interchemical Corporation introduced the first mechanically engraved metering roller, known as anilox roller [2]. Initially used for packaging due to its fast-drying properties, flexography was officially named in 1952, marking a milestone in its evolution [3]. The first Flexographic Forum was celebrated in 1957, and the next year, Flexographic Technical Association was founded. The publication of “Flexography principles and practices” contributed to standardizing and making advances in industry [2]. In 1964, the introduction of the scrapper, which replaced the rubber roller, revolutionized ink metering. The advent of process color printing by DuPont in 1965 significantly enhanced the operational capabilities of flexography. During the 1980s, advancements in the fabrication of photopolymer plates culminated in products that exhibited increased strength and simplified production processes. This era was also marked by the introduction of laser-engraved ceramic anilox rollers, which notably enhanced both ink distribution and consistency. The commercialization of UV pigments in the late 1990s highlighted the emphasis of this era on quality and efficiency, thereby establishing the groundwork for contemporary flexographic printing. Innovations in plate exposure technology were manifold, particularly with the advent of LED exposure units. These developments facilitated a more meticulous regulation of the exposure process, culminating in the production of plates of superior quality. The introduction of flatbed dot technology transformed print quality by optimizing ink transfer and dot stability [2] (Figure 1). Throughout the 20th century, the evolution of technology was marked by advancements in photopolymer plates and UV inks, thereby augmenting its versatility.

The digital revolution in flexography began with the introduction of Laser Ablation Mask Systems (LAMSs) in the late 20th century, improving the precision and efficiency of plate production [1]. The optimization of every processing stage of photopolymer printing plates intended for thermal development has been investigated [4]. However, the launch of the Kodak FLEXEL NX system in 2008 marked a turning point. With its flat-top dot technology, FLEXEL NX elevated flexographic print quality to levels comparable to gravure and offset [5]. Its ability to reproduce fine details and a wide range of colors expanded the possibilities of flexography, establishing it as a leader in digital flexographic printing [6].

**Figure 2:**
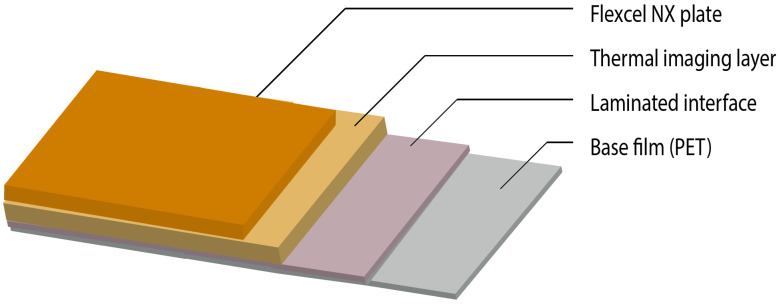
Schematic of the FLEXEL NX plate layers and their functions.

The FLEXEL NX plate is composed of several layers (Figure 2), each with a specific role in the printing process (Table 1):

Printing technologies facilitate rapid and cost-effective prototyping and the production of flexible sensors, which can be embedded in various materials and wearables [10]. Flexographic printing is a form of relief printing where a flexible printing plate transfers ink onto a substrate. The resolution of flexographic printing has been improved by adding engineered nanoporous microstructures, aligned carbon nanotubes, to reach the creation of features at the nanometer scale [11].

Flexography has been adapted for microfluidic applications by modifying the ink, substrate, and printing conditions. Therefore, the flexibility in material choice and substrate compatibility of flexography allows for the creation of diverse microfluidic architectures, including channels, valves, and sensors.

Microfluidics has revolutionized various fields such as biology [12], biomedical diagnostics [13], chemical analysis [14], and environmental monitoring [15] due to its ability to manipulate small volumes of fluids effectively. It is noteworthy that the COVID-19 pandemic has increased the need for microfluidic devices that can be used for quick diagnosis at the point of care [16]. This has led to a search for manufacturing methods that are fast, reliable, and affordable. The conventional methods of microfluidic device mold fabrication are soft lithography [17], multi-photon lithography [18] using SU-8 epoxy-based photoresist, CO_2_ laser ablation [19], computer numerically controlled machining [20], 3D printing [21], a combination of 3D printing/CNC micro-milling [22] and others. Nevertheless, these methods require clean room facilities, expensive equipment, and high maintenance costs. Consequently, there is a growing need for inexpensive ways to create microfluidic devices [23]. Generally, the more resolution the method provides, the higher the cost it implies (Table 2). For instance, the most common photoresist used to produce microfluidic molds is SU-8, which facilitates the development of microstructures exhibiting a high degree of resolution (~1 μm) [24]. Nevertheless, its application is constrained to laboratories equipped with costly apparatus for the generation of masks and the execution of lithographic techniques [25]. This makes the manufacture of high-resolution microfluidic devices for small-to-medium-sized research laboratories impossible or unaffordable. Following its initial utilization as an unconventional manufacturing technique, FMold exhibits the potential to evolve into a prevalent methodology capable of exceeding traditional manufacturing practices

After the market launch of Kodak FLEXCEL NX Ultra Solution by Kodak in 2019 [34], it has been widely and globally used in the packaging industry [35]. This massification in its use has made it available in practically all higher-income and lower-income countries (Supplementary Table S1). The process of FMold manufacturing has become significantly more attainable in comparison to photolithography, as there currently exist more than 400 enterprises dispersed across 60 nations that provide FMold fabrication services [25]. As an example of costs, an FMold of 400 cm^2^ (20 cm × 20 cm × 1.14 mm) polymer print with a resolution of up to 10 microns can be obtained for EUR 12.17 in Italy [36], USD 105 in the United States [37], USD 30 in Ecuador and Colombia, and USD 28 in Argentina.

**Figure 3:**
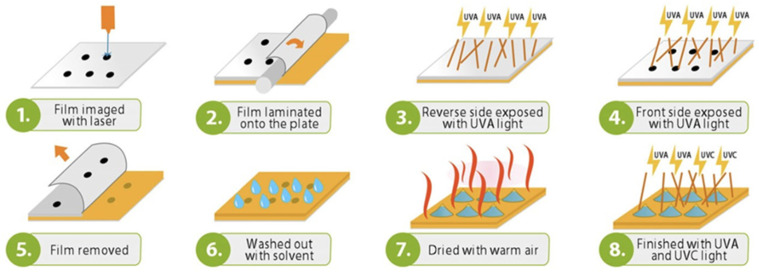
FMold method fabrication [25]. (**1**) The design is transferred to the film through 2400 ppi laser. (**2**) The film is placed onto the plate. (**3**) The reverse side is exposed to UVA at 0.45 J. (**4**) The front side is exposed to UVA at 19 J during 360s. (**5**) The film is removed. (**6**) Washing process with PROSOL N-1 solvent. (**7**) Air-drying process at 50 °C during 30 min. (**8**) Finally, the front side is exposed to UVC at 10 J during 17 min and UVA at 4 J during 2 min. License number: 5891431414309. License date: 17 October 2024. Licensed content publisher: Wiley and Sons.

As shown by Bourguignon et al. 2018 [25], the fabrication of the master mold using the polymer called FMold (Figure 3) requires the microchannel network designed in Layout Editor software. This conceptual framework is subsequently transposed onto a DITR film via a 2400 ppi infrared laser apparatus. The film is then affixed to an unexposed flexographic printing plate, which undergoes exposure to UVA radiation at an energy of 0.45 J on the reverse side and 19 J on the front side over a duration of 360 s, with UVA exposure time on the reverse side being subject to variation. Upon the removal of the film, the plate is subjected to a washing procedure utilizing PROSOL N-1 solvent at a flow rate of 360 mm min^−1^, followed by an oven-drying process at a temperature of 50 °C for a duration of 30 min. Ultimately, the plate is exposed to 10 J of UVC light for 17 min and to 4 J of UVA light for 2 min on the front side. An analogous methodology is employed to fabricate the FMold for the PDMS-floor replica, with the exclusion of the layout design phase. The resultant FMold is subsequently coated with an ultrathin SiO_2_ film through the technique of plasma-enhanced chemical vapor deposition (PEVCD). The authors utilized a custom-built PECVD reactor that provides a continuous glow discharge of 900 V, characterized by capacitive coupling and impedance matching.

The vacuum chamber constituted a Pyrex glass tube with a length of 80 cm and a diameter of 15 cm. Hexamethyldisilazane (HMDS) sourced from Dow Corning was utilized as the precursor monomer. The operational gas (O_2_) was introduced at the remote end of the chamber, thereby enabling vapor ionization within the discharge zone for the synthesis of SiO_2_ coatings. The deposition process was conducted under specific parameters: an O_2_ flow rate of 8 mL s^−1^, a gas pressure of 1 mbar, and an exposure duration of 3. The non-treated FMold is designated as “FMold,” whereas the SiO_2_-coated mold is referred to as “FMold-T”.

**Figure 4:**
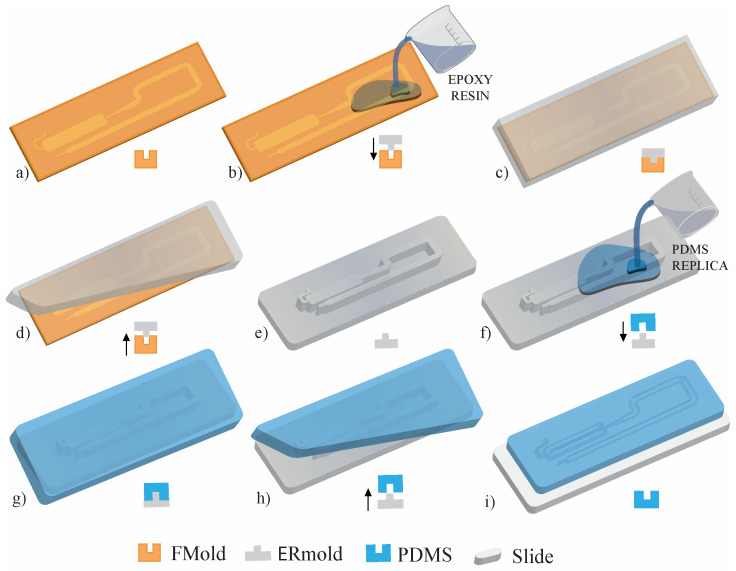
PDMS microdevice fabrication: (**a**) A photopolymeric flexographic master mold (FMold) is utilized. (**b**,**c**) The epoxy resin is applied onto the FMold and subsequently cured at a temperature of 25 °C. (**d**,**e**) Following a duration of 72 h, the ERmold is detached, resulting in the formation of the male mold. (**f**,**g**) The PDMS is applied onto the ERmold and cured at a temperature of 40 °C for an overnight period. (**h**) The PDMS replica is subsequently removed from the mold. (**i**) Fluidic connection ports are created by punching, after which the replica is irreversibly bonded to a glass wafer through exposure to plasma. The PDMS replica is designed in accordance with a microdevice intended for microdroplet generation. Down arrows indicate the liquid polymer pouring whereas up arrows show solid polymer removal. License date: 21 October 2024. Licensed content publisher: Elsevier. License number: 5893781316802.

This was the first time that photopolymer flexographic printing was used for microfluidic device manufacturing [25]. An innovative and cost-efficient method for fabricating polydimethylsiloxane (PDMS) microdevices utilizing a photopolymer printing plate referred to as FLEXCEL as the master mold (FMold) has been reported [25]. This methodology facilitates the generation of numerous devices from a single master, accomplishing a minimum channel dimension of 25 μm, structural heights ranging from 53 to 1500 μm, and spatial extents reaching up to 1270 × 2062 mm^2^, thereby exceeding those achieved by current techniques. Scanning electron microscopy, atomic force microscopy, and profilometry were employed to comprehensively characterize both the FMold and its PDMS replicas. In order to enhance the ease of PDMS replica detachment from the FMold, a SiO_2_ coating was applied, and the results demonstrate a remarkable replication fidelity from the FMold to the PDMS replicas. The mold exhibits the capacity for repeated utilization through the acquisition of reliable replicas, as there is no risk of delamination due to the mold and the structure being integrated into a single piece. Additionally, the reusability of the FMold has been evidenced, with the successful production of up to 50 PDMS replicas without any discernible degradation of the mold. However, the SiO_2_ coating made by PECVD is not accessible in the majority of research laboratories. Therefore, the massification of the use of FMold came in 2019, when female FMold channel design was directly transferred to an epoxy resin as published by Olmos et al. 2019 [27]. It was the first time that FMold was applied as a female mold to transfer channel architecture to fabricate male epoxy resin molds to obtain PDMS microfluidic devices as depicted in Figure 4. This innovative approach eliminates the necessity for SiO_2_ coating and facilitates the production of male epoxy resin molds incorporating structures with diverse topologies, thereby streamlining methodology and rendering microfluidic mold fabrication attainable for laboratories in lower-income countries due to low cost, simplicity, and scalability, which is congruent with Sustainable Development Goal (SDG) 10, “Reduce inequality within and among countries”.

Since then, this technique has been utilized in numerous publications by different universities and research groups worldwide, for example, in enhanced oil recovery to prepare rock-on-a-chip models [38], droplet generation and storage [39], suspension cell culture [28], monoclonal antibody production [40], complex cell differentiation pattern creation [41], mixing solutions by automatic feedback control [42], phage screening [43], drug screening [44], cancer stem cell culture [45], droplet storage for gene editing [46], culture of spheres derived from cancer stem cells isolated from veterinary patient tumors [47], drug screening against *Trypanosoma cruzi* [44], and hybrid microchannel-solid micropore device fabrication [48].

The flexographic method offers several advantages (Table 3), encompassing (a) economic efficiency, (b) extensive accessibility, (c) the absence of requirement for cleanroom environments, (d) rapid mold production, (e) elevated mold durability, (f) minimal surface roughness of structures, and (g) the capability for the high-throughput manufacturing of epoxy resin molds and PDMS replicas with accurate replication [25,27].

**Figure 5:**
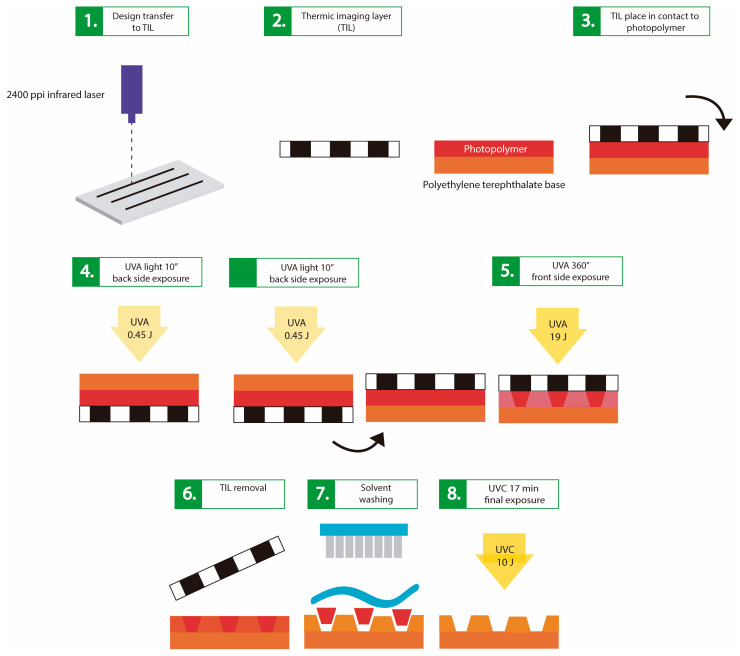
Schematic representation of the fabrication procedure for the FMold mold utilizing the FLEXEL NX photopolymer sheet and the thermal imaging layer (TIL): (**1**) The design is transposed onto the thermal imaging layer (TIL) employing a 2400 ppi infrared laser apparatus. (**2**,**3**) The TIL is positioned over the photopolymer plate, and (**4**) the reverse side of the photopolymer plate is subjected to UVA radiation at an energy of 0.45 J for a duration of 10 s, with a segment of the photopolymer being occluded by a mask on the reverse side; subsequently, the reverse side of the photopolymer plate is exposed to UVA light at 0.45 J for 10 s, and (**5**) the front side is then exposed to the UVA light at 19 J for a total of 360 s. (**6**) Following this, the TIL is subsequently removed. (**7**) The photopolymer plate undergoes a washing process utilizing PROSOL N-1 solvent at a flow rate of 360 mm·min^−1^, and it is subsequently dried in an oven at a temperature of 50 °C for 30 min. (**8**) Finally, the front side of the photopolymer layer is exposed to UVC radiation at 10 J for a duration of 17 min and to UVA radiation at 4 J for an additional 2 min.

The subsequent research investigation illustrated the application of FMold in the fabrication of multi-level molds and the transference of these structures to PDMS replicas, wherein the varying heights on the PDMS mold were regulated by modulating the UVA exposure duration and the channel widths [28] as elucidated in Figure 5. This study presented a cost-effective method for fabricating photopolymer molds with multi-level microstructures. The molds were characterized using various microscopy techniques, and the results showed that the method can produce structures with a variety of depths and heights. The potential applications of this method were demonstrated by creating a microfluidic device for cell culture and proliferation.

In addition, photopolymers have the capacity to facilitate the fabrication of intricate three-dimensional patterns through the utilization of a simple grayscale mask in conjunction with a FLEXCEL flexographic photopolymer. When the exposure duration is consistently maintained across the entirety of the plate, the varying elevations incorporated within the mold are archived via the grayscale mask [41]. This methodology is economically advantageous and possesses the capability to generate a diverse array of three-dimensional topographies and structures. The researchers illustrate the efficacy of this approach by producing a sophisticated 3D pattern on a PDMS mold. Subsequently, they employ the mold to fabricate a stamp intended for microcontact printing applications. The stamp is utilized to imprint a specific arrangement of human-induced pluripotent stem cells onto a cell culture substrate. Such a technique may prove beneficial for the development of complex three-dimensional patterns applicable to numerous fields, encompassing microfluidics and microelectromechanical systems.

## 2. Applications of FMold in Microfluidics

### 2.1. Chemical Applications

**Figure 6:**
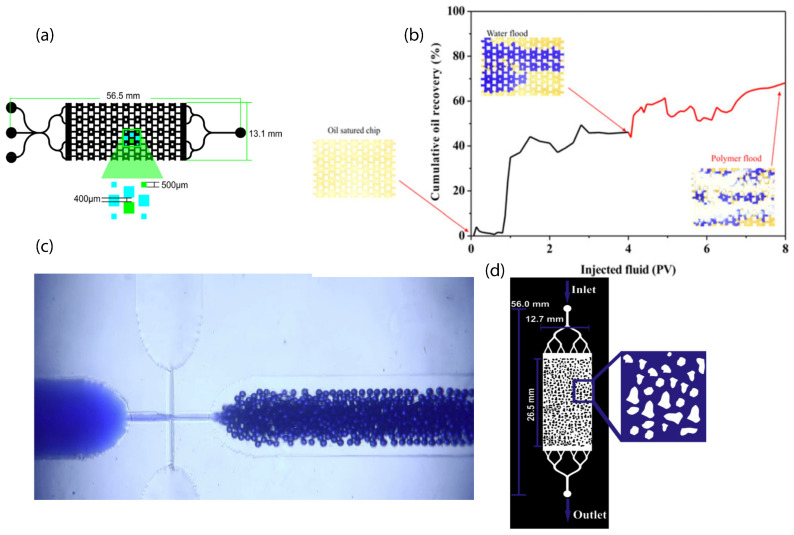
(**a**) The design of channel architecture for EOR experimental investigation. (**b**) An analysis of oil recovery, focusing on cumulative oil recovery as a function of pore volume (PV), accompanied by visual representations of oil-saturated, dyed water flood, and polymer flood phenomena [25]. License number: 5898940589195. License date: October 2024. Licensed content publisher: Wiley and Sons. (**c**) The generation of microdroplets utilizing a microfluidic flow-focusing device. Representative images obtained through optical microscopy of the microdroplet (magnification: 5×) [27]. License date: 21 October 2024. Licensed content publisher: Elsevier. License number: 5893781316802. (**d**) Microfluidic device with random network design. An enlarged depiction of the pores/grains within the porous media is illustrated in blue. The microfluidic porous media devices were synthesized using polydimethylsiloxane (PDMS) and glass substrate [38]. License date: 21 October 2024. License number: 5893790398924.

FMold has been used to manufacture microdevices with specific channel architecture that reflect soil portal throats for enhanced oil recovery (EOR) assays [25]. Briefly, the microdevice shown in Figure 6a was saturated with oil at 1 mL·h^−1^ flow rate until it became entrapped within the pore spaces. Afterwards, dyed water (blue) was injected at two flow rates, 0.5 and 2 mL·h^−1^, to simulate primary oil recovery, and finally polyacrylamide polymer solution (1000 ppm) was administered at 0.5 and 1 mL·h^−1^ to extract additional oil, thereby simulating secondary oil recovery. The quantification of the recovered oil was conducted through image analysis with Image J 1.54f. The variation between the initial condition of the black pixels and the final condition was interpreted as indicative of oil recovery. The cumulative oil recovery achieved was 67%, which correlates with the number of poral volumes utilized for each substance (Figure 6b).

Furthermore, FMold was employed to fabricate a microfluidic device designed for a droplet generator. The flow-focusing microchannel architecture was transposed from the FMold to an epoxy resin mold, subsequently allowing for its replication in PDMS [27]. This methodology yielded a microdroplet generator capable of producing monodispersed droplets (Figure 6c) from 47.4 μm to 63.1 μm with a standard deviation lower than 4%, thereby demonstrating that the microfluidic device was proficient in generating droplets of controlled dimensions, which holds considerable significance in numerous chemical [52], biomedical [53], and industrial applications [54].

**Figure 7:**
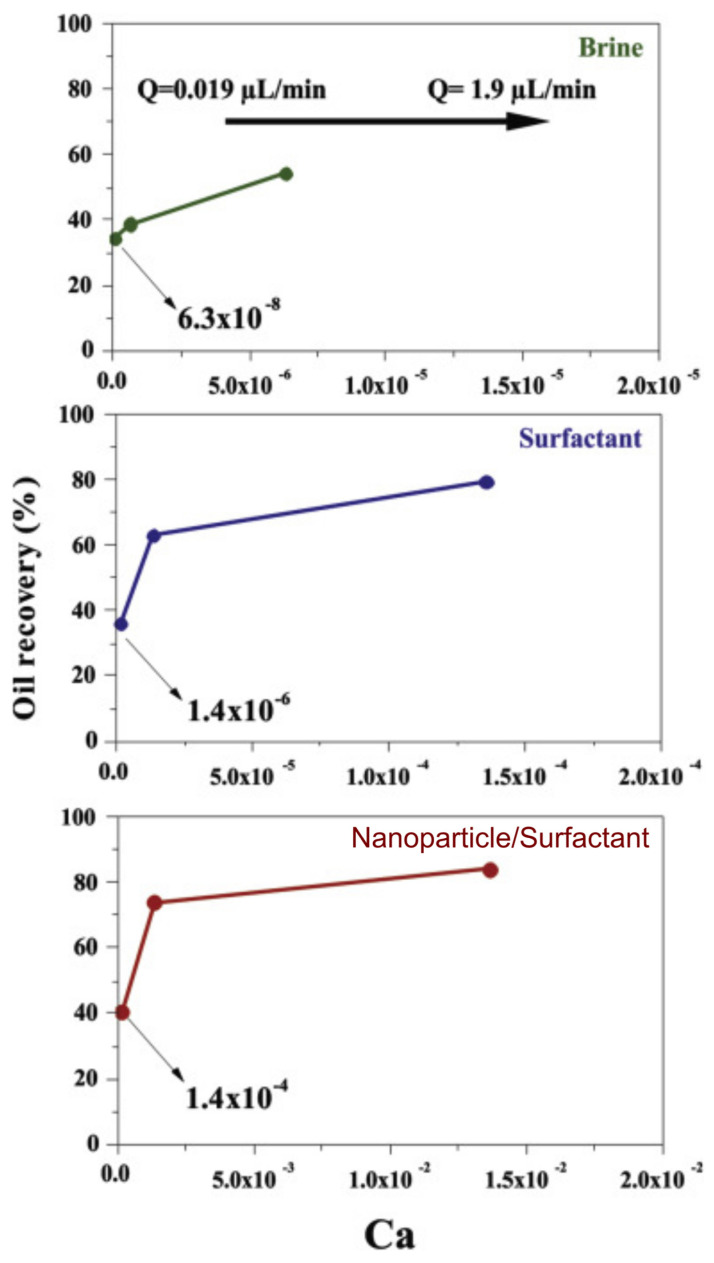
The extraction of oil as a function of capillary number is analyzed for various flooding techniques, including waterflooding, surfactant flooding (utilizing a surfactant combination of S1 and S2) and nanoparticle/surfactant flooding (incorporating a nanofluid composed of magnetic iron core–carbon shell nanoparticles alongside the surfactant mixture of S1 and S2) at injection rates of 0.019 μL·min^−1^, 0.19 μL·min^−1^, and 1.9 μL·min^−1^ [38]. License date: 21 October 2024. License number: 5893790398924.

In addition, FMold has also been useful for the creation of a microfluidic device applied to assess the effect of the concurrent application of a surfactant mixture and magnetic iron core–carbon shell nanoparticles on oil recovery through a microfluidic analysis based on the rock-on-a-chip technology [38]. The displacement experiments encompassed waterflooding, surfactant flooding, and nanoparticle/surfactant flooding, all conducted utilizing PDMS (polydimethylsiloxane)-glass microdevices of the random network type as illustrated in Figure 6d. The findings indicated that the simultaneous application of nanoparticles and surfactants could serve as a cost-effective alternative in EOR processes. This investigation employed microfluidic devices to replicate reservoir conditions and scrutinized the influence of varying injection rates on oil recovery. The authors observed that the synergistic application of nanoparticles and surfactants substantially enhances oil recovery in comparison to conventional waterflooding techniques. This enhancement can be attributed to multiple factors, including the modification of the wettability of the porous media, the reduction in interfacial tension, the increase in viscosity, and the alteration of flow dynamics. Furthermore, the study underscores the critical importance of optimizing injection rates to maximize oil recovery, with elevated injection rates generally resulting in improved outcomes. The influence of the injection rate was systematically evaluated, varying at distinct rates of 0.019 μL·min^−1^, 0.19 μL·min^−1^, and 1.9 μL·min^−1^. The ultimate oil recovery was maximized at the injection rate of 1.9 μL·min^−1^. The escalation of the injection rate augmented the viscous force of the fluid, which in turn increased the capillary number (Figure 7). Specifically, at an injection rate of 1.9 μL·min^−1^ during nanoparticle/surfactant flooding, the capillary number reached 1.4 × 10^−2^, culminating in an oil recovery rate of 84%.

These findings imply that the incorporation of nanoparticles and surfactants in EOR processes may represent a cost-effective strategy to enhance oil extraction.

**Figure 8:**
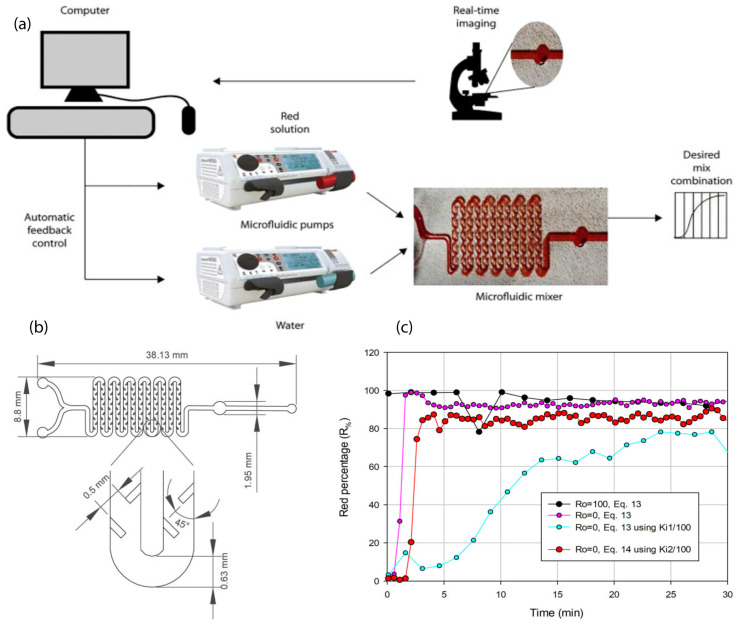
(**a**) Schematic representation of the experimental configuration employed for mixing solutions through the automatic regulation of pressure-induced flows, utilizing control theory and real-time image capture. (**b**) Configuration of the microfluidic device utilized for color mixing investigations. (**c**) Regulation of red percentage (integrative control) with respect to variable integral gain. The desired red value R_%*jdes*_ that was evaluated was 80, and the preliminary red flow is $v_{r}^{0}=0.01\;\mu L/min.$ Modified from [42]. Published under an exclusive license by AIP Publishing. © 2022 Author(s).

In addition, flexographic material was applied to produce a microfluidic micromixer (Figure 8b) device that enabled the automatic regulation of the decrement in color percentage upon the injection of dye, by employing pressure-driven flows to mix solutions through the application of control theory and real-time image capture for the inaugural instance [42]. The configuration comprises a microfluidic device interconnected with two syringe pumps, which deliver dye solution (red) and water at a flow rate such that the sum of both flows remains invariant (Figure 8a). The regulation and stabilization of the microfluidic system were successfully attained for dye concentrations exceeding 60%. The flow of the mixture attains a red concentration percentage that aligns as close as possible with the red percentage specified by the user. Initially, the system is assessed devoid of any feedback control to elucidate how varying input flow rates influence the output. This process generates a series of calibration curves. These curves are subsequently employed to ascertain the control parameters pertinent to the system. For the purpose of automatic control, two methodologies were investigated: integrative control featuring variable integral gain and constant integral gain. Figure 8c illustrates the outcomes of the control of red percentage against time and demonstrates that the saturation curves commencing from zero percent (i.e., $R_{\%}^{0}=0$) are those that most accurately approximate the desired value.

Finally, an integrative control strategy is executed, leveraging both fixed and variable gains to optimize the system’s responsiveness. The regulation of color percentage could serve as a pivotal tool modulating other parameters such as concentration applied to cell culture and the control of alkalinity (pH) of solutions within microfluidic devices.

### 2.2. Biomedical Applications

#### 2.2.1. Suspension Cell Culture, Jurkat Cell Line, and Healthy Donor T-Cells

**Figure 9:**
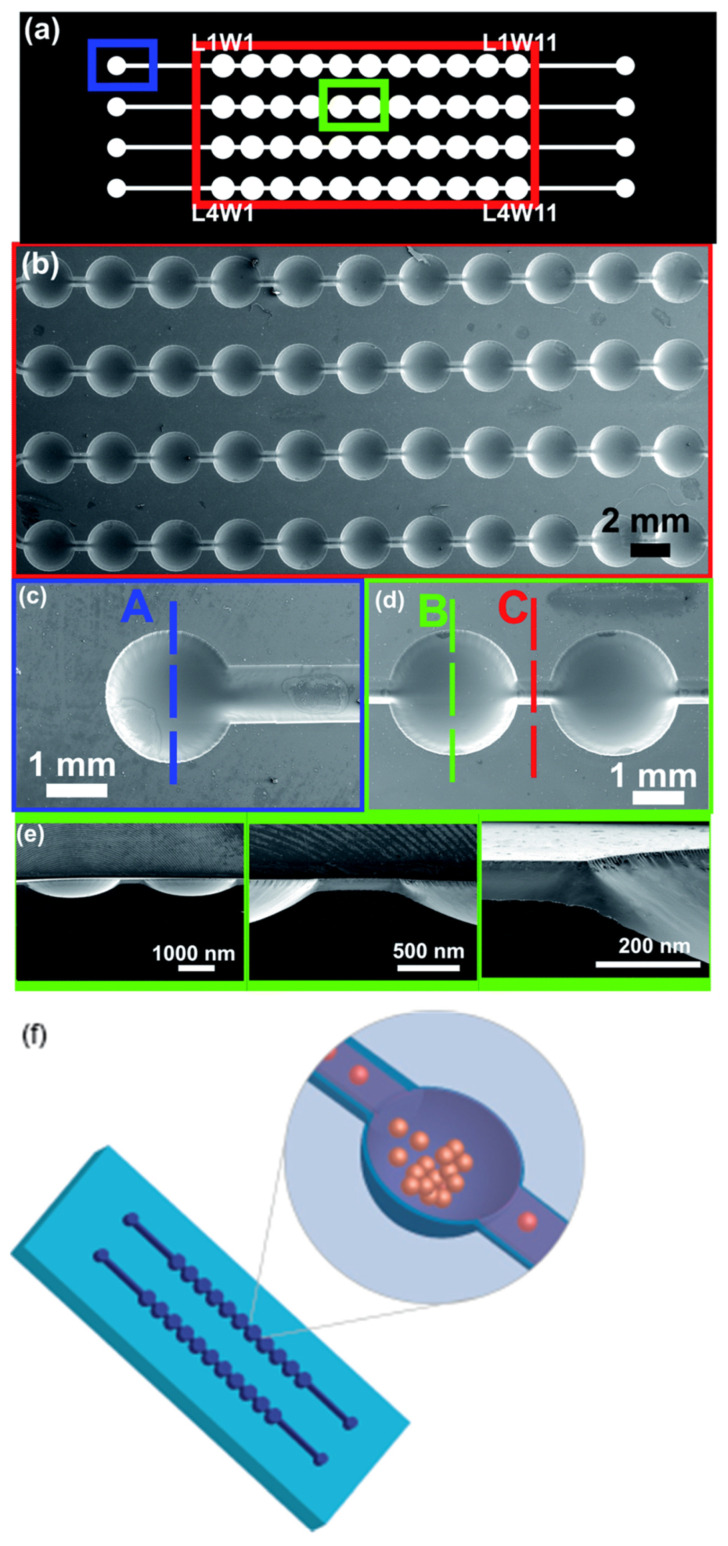
(**a**) The network of microchannels within the microfluidic device, (**b**) SEM image depicting the wells, (**c**) SEM images of zone A at an increased magnification, (**d**) SEM images of zone B and C at an augmented magnification, and (**e**) SEM images illustrating the cross-sectional view of the mold. Conditions for the fabrication of the photopolymer mold are as follows: firstly, UVA exposure duration on the reverse side: 10 s, exposure duration on the front side: 360 s; secondly, UVA exposure duration on the front side: 2 min, UVC exposure duration on the front side: 17 min. Modified from [28] with permission from the Royal Society of Chemistry. (**f**) Schematic showing microchambers that gently trap healthy donor T-cells. Used under Creative Commons Attribution (CC BY) license [55].

Multi-level design shown in Figure 9a and SEM imaged in Figure 9b enabled harboring Jurkat cells at the deepest zone of each well (B zone, as marked in green in Figure 9c), allowing cell proliferation. This methodological approach enabled the comprehensive characterization of the development of suspension cells, as the distinct levels allowed for the gentle entrapment of cellular aggregates within the wells while simultaneously permitting the renewal of the culture medium (zone C, indicated in red in Figure 9c) without adversely impacting cell proliferation. A volume of 65 μL of Jurkat cell suspension was introduced into each channel of the microfluidic device, resulting in the dispersion of cells throughout the wells. Thereafter, the cells underwent sedimentation due to gravitational forces and congregated within the B zone. This phenomenon underscores the efficacy of the multi-level configuration, as cells are immobilized at the bottom, while the upper zone facilitates the flow of the medium. The microchannel networks devised for the fabrication of the multi-level microstructures (Figure 9e) were produced by modulating UVA exposure duration and by adjusting the channel width within the design [28]. The methodology developed exhibits significant potential across various domains of microfluidics, particularly in the cultivation of non-adherent cells such as Jurkat cells and T-cells derived from healthy donors [43], to investigate T-cell expansion within microfluidic devices (Figure 9f) and to generate tumor spheroids; different depths have been examined, and the optimal microwell depth was identified as 300 µm for the uniform culture of tumor spheroid [22].

#### 2.2.2. Monoclonal Antibody Production

**Figure 10:**
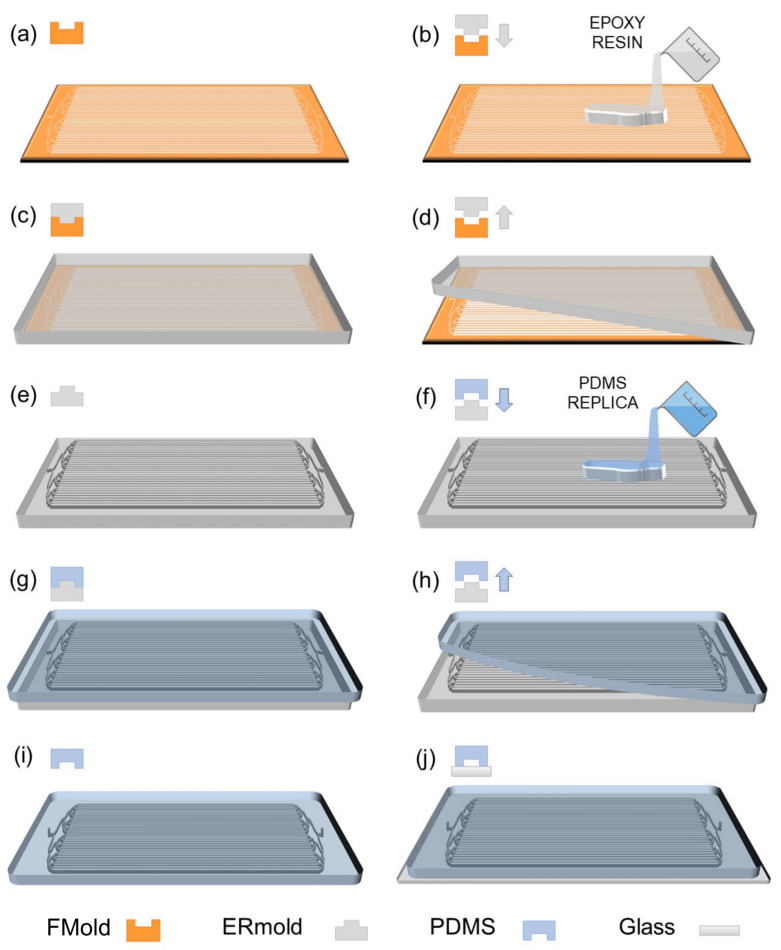
The fabrication process of the PDMS LM bioreactor device: (**a**) a female patterned photopolymeric flexographic master mold (FMold) is utilized; (**b**) an epoxy resin layer is meticulously cast upon the female FMold; (**c**) the epoxy resin undergoes a curing process at 25 °C for a duration of 72 h; (**d**) the cured epoxy layer is subsequently peeled away; (**e**) the male ERmold is now created; (**f**) the PDMS replica is cast onto the male ERmold; (**g**) the PDMS layer is cured at 40 °C overnight; (**h**) the female PDMS replica is then detached; (**i**) fluidic connection entry and exit ports are precisely punched into the female PDMS; (**j**) the PDMS replica, featuring the design of the bioreactor microfluidic channels, is irreversibly bonded to glass wafer through plasma exposure. Down arrows indicate the liquid polymer pouring whereas up arrows show solid polymer removal. Reused under the terms and conditions of the Creative Commons Attribution (CC BY) license [59].

Commercially, monoclonal antibody (mAb) production takes place in 5 to 20 m^3^ bioreactor tanks with mammalian cells via shaking suspension culture, which is conducted in either fed-batch or perfusion modes [56]. A promising approach to enhance the comprehension and regulatory oversight of mAb production in bioreactors is the miniaturization of the platform through the utilization of microfluidic devices [57] as microfluidics facilitates the exploration of the involved factors that govern mAb production. The photopolymer flexographic master mold provided the opportunity to manufacture a large-area microfluidic bioreactor (LM bioreactor) (Figure 10) designed for mammalian cell culture, functioning under laminar flow and perfusion conditions. Notably, this bioreactor was patented [58]. The LM bioreactor was integrated with a syringe pump system to facilitate the perfusion of culture media, while cell cultures were analyzed through photomicrograph imaging. The CHO-ahIFN-α2b adherent cell line, which produces the anti-hIFN-α2b recombinant scFv-Fc monoclonal antibody (mAb) intendent for the treatment of systemic lupus erythematosus, was cultured within the LM bioreactor; notably, for the first time, the recombinant monoclonal antibody synthesized in a microfluidic device demonstrated superior functional activity compared to antibodies generated under conventional culture conditions [59].

#### 2.2.3. Complex Cell Line Differentiation

FLEXCEL photopolymer allows the transfer of patterns to PDMS replicas with considerable consistency. This characteristic has been employed in the fabrication of a milli-bioreactor, which was subsequently validated through the culturing of human-induced pluripotent stem cells (hiPSCs) over a duration of 5 days [40]. This innovative microfluidic bioreactor incorporates diminutive pillars (passive control) to shield cells from damage inflicted by bubbles. The authors proposed an approach utilizing computational fluid dynamic simulations to characterize the designs prior to the fabrication of the device. The evaluation included a combination of a single-chamber design with 3 pillars and a design featuring 10 pillars, with channel heights measuring 270 µm and 2 mm, respectively. The 2 mm height design proved beneficial in sustaining a stable cell culture, as it allowed for the accommodation of bubbles at the chamber’s upper section, while no alterations in cell viability were detected beneath the bubble. This microfluidic platform is not only straightforward to construct but also cost-effective to produce and user-friendly with a variety of cell types. The pillar-based milli-bioreactor design offers effective bubble management, sufficient working volume, capabilities for real-time monitoring, and high yield of cells for extended cultivation periods. This addresses significant challenges in microfluidic cell culture, thereby rendering it a valuable instrument for both cell cultivation and analysis.

**Figure 11:**
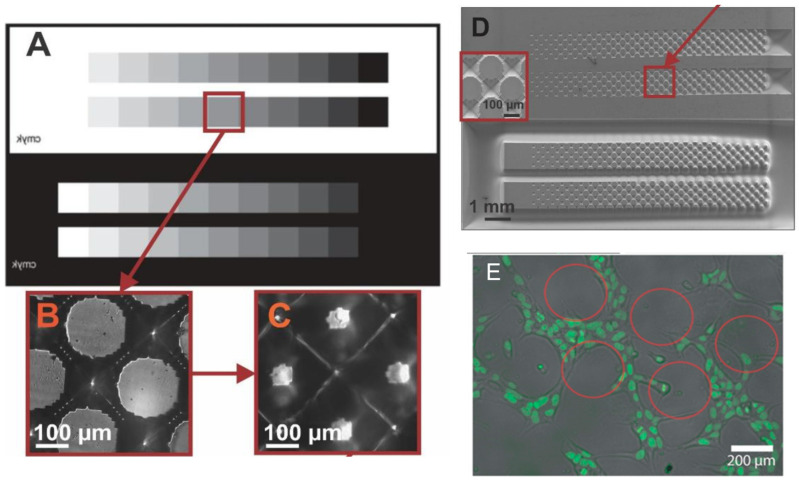
(**A**) Design of the gray scale mask. (**B**) Optical representation of the pattern within the photopolymer flexographic mold (FMold). (**C**) Optical depiction of the resultant pattern within the epoxy resin mold (ERmold). (**D**) SEM image of the PDMS stamp, with the inset providing a magnified view of the corresponding pattern derived from the mask. (**E**) Brightfield and overlay images illustrating the selective adherence of hiPSCs to the segments where the pattern was delineated. The red outlines present the dots of the patterns established through the microcontact printing technique; cellular growth was observed around the dots. Modified from [41]. Used under the terms of the Creative Commons Attribution License 4.0 license.

Furthermore, the FLEXCEL photopolymer enabled the creation of an innovative lithography technique predicated on grayscale patterns printed in an FMold and subsequently transferred to epoxy resin in order to replicate them in a PDMS stamp, thus achieving microprint pattern structures. This methodology has been employed to generate intricate 3D patterns utilizing a grayscale mask (Figure 11A), which was designed with Layout Editor 20220423 software applying the amplitude-modulated approach, a traditional screening technique that modifies the dot size while maintaining a constant distance between the dots. Upon obtaining the FMold (Figure 11B), the structures were transferred to an epoxy resin mold (Figure 11C) and subsequently to a PDMS replica (Figure 11D) for microcontact printing, thereby generating a corresponding pattern of hiPSCs on the cell culture plates that had been treated with reagents conducive to adhesion. The geometry of the patterns can be regulated by adjusting the layout and grayscale of the stamp patterns [41]. For the seeding of hiPSCs, 1 × Geltrex was applied to the previously treated surface. The Geltrex was allowed to set for 1 h, after which 10,000 cells·mL^−1^ were seeded.

Indeed, microcontact printing can prove highly advantageous in the creation of specific patterns that facilitate complex differentiation in cell lines such as hiPSCs (Figure 11E).

**Figure 12:**
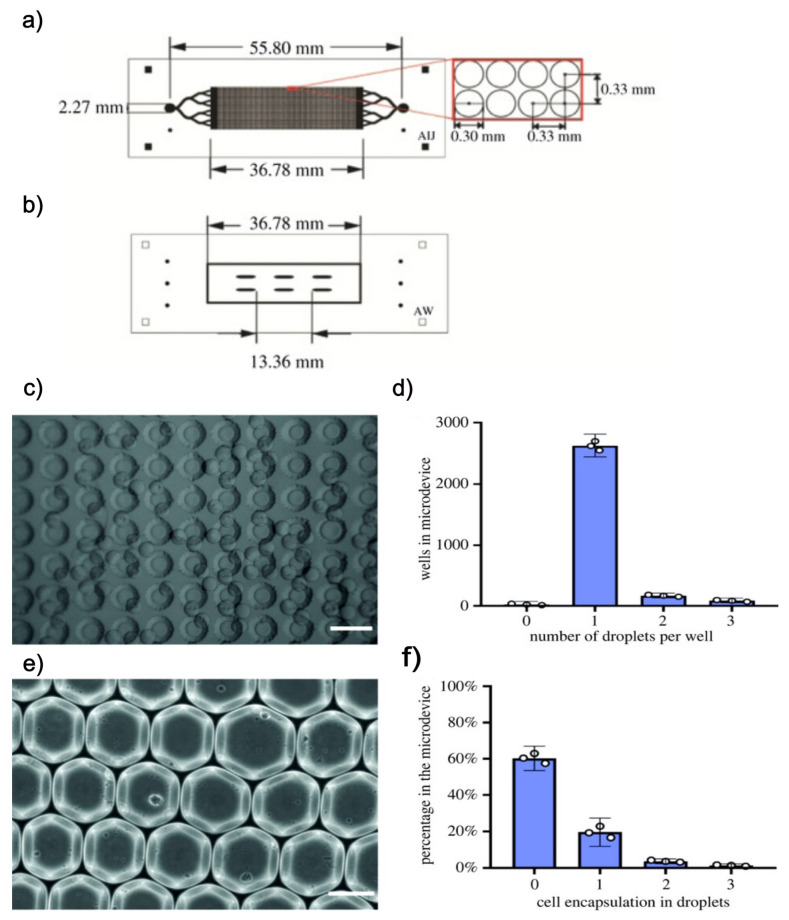
Storage device for microdroplets that encapsulates transfected single cells comprises two layers. (**a**) The initial layer comprises the storage wells. (**b**) The subsequent layer is constituted by the inlet channels along with the support pillars. (**c**) Representative visual depiction of droplets retained within the micro-storage device. (**d**) The quantification of droplets retained in each well of the micro-storage device. The white points signify the mean values for each replicate (N = 3). (**e**) A representative visual depiction of cells inside that traverse through the outlet channel of the forming device. (**f**) The bar graph illustrates the efficiency of capturing a single cell per droplet. The white points denote the mean value for each replicate (N = 3). The scale bars are representative of 100 μm. Modified from [39]. Used under the terms of the Creative Commons Attribution 4.0 license.

Other applications of FMold consist of the manufacturing of a microdroplet storage device (Figure 12a,b), which is a part of a microfluidic transfection setup that reported an 80% transfection efficiency of hiPSCs. This research approach employs PDMS multi-level architecture, which facilitates the categorization of droplets based on both manufacturing dimensions and depth. This technique exhibits significant versatility, enabling the fabrication of storage devices for droplets with diameters from 30 μm to 3 mm [39]. The resultant multi-level microdevice is meticulously engineered with thousands of wells that serve as reservoirs for droplet accumulation, thereby supporting cell culture and the real-time observation of cell-to-cell transfection processes and cell viability over time. High efficiency of the droplet storage device was determined as it was able to capture a single droplet per well, which is exceedingly advantageous for image analysis and real-time cellular detection. In Figure 12c, the wells of the storage microdevice were visualized, effectively capturing the droplets containing cells within. Furthermore, the extent of cellular encapsulation per droplet was systematically assessed. Figure 12d presents cells encapsulated within monodisperse droplets generated by the system. According to the Poisson distribution, it was observed that the predominant proportion of droplets did not contain any cells, achieving an encapsulation efficiency of 22% for single cells (Figure 12f). Given that the storage device is capable of accommodating droplets of diverse sizes and volumes while facilitating rapid and cost-effective manufacturing, it is regarded as superior to other analogous systems. The multi-level storage microdevice permits the operator to visualize and ascertain the positioning of each cell throughout the transfection process. The implementation of single-cell transfection via the microfluidic droplet methodology has been demonstrated to be exceptionally efficient and cost-effective in comparison to alternative methodologies.

#### 2.2.4. Phage Screening According to Affinity

**Figure 13:**
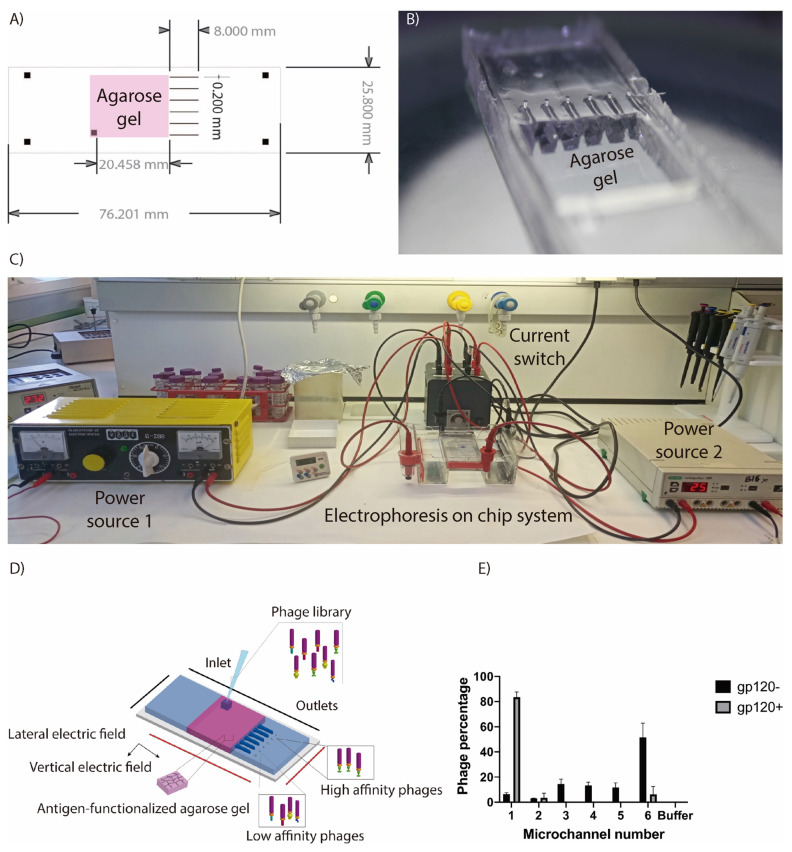
(**A**) The design of the microdevice includes an inlet for sample introduction and six outlet channels designated for phage collection. (**B**) The PDMS microdevice is fabricated from a master mold, followed by the adhesion of the PDMS replica to a glass slide. Agarose gel was synthesized within the central reservoir. (**C**) An image depicting the complete system designed for phage selection via electrophoresis featuring the electrophoresis chamber and the integrated microdevice, along with the energy sources and current switch equipment. (**D**) A schematic illustration of the microdevice; phages are introduced through the inlet and traverse the agarose gel functionalized with target antigens, subjected to two alternating electric fields, namely, lateral (X) and vertical (Y) fields. The phages can be sorted based on their antigen-binding affinity and subsequently collected at the six outlets indicated in this schematic representation. (**E**) Visualization of S20-phage migration within the microdevice, in functionalized agarose gel both in the absence (−) or presence (+) of gp120 protein. The percentage of the total quantity of phages recovered in each channel is presented. Approximately 2.5 μL of S20-phages (10^9^ PFU·mL^−1^) is used in each experimental trial [43]. License number: 5893800577854. License date: 21 October 2024. Licensed content publisher: John Wiley and Sons.

FMold has been utilized to produce a PDMS microdevice provided with six channels (Figure 13A) and a center chamber for agarose gel (Figure 13B) to separate and classify phages from a library according to their affinity to the protein of interest by electrophoresis [43]. An agarose gel was modified with the protein (HIV-1 gp120 or EBOV-GP) in order to identify phages exhibiting high, intermediate, and low affinity that are allocated in distinct microchannels through the application of alternative lateral (X) and vertical (Y) electric fields (Figure 13C–E). Significant endeavors have been undertaken to formulate therapeutic interventions and preventive measures against the severe Ebola virus (EBOV) disease (EVD) with a focus on targeting the EBOV-GP. A combinatorial library of EBOV derived from B cells isolated from an immunized donor was processed through our microdevice to isolate phages with high affinity for EBOV-GP. This methodology serves as an efficient mechanism for the separation of antigen-bound phages from unbound counterparts within such libraries. As illustrated in Figure 13E, when the agarose is functionalized with GP120 protein, a predominant recovery of phages with high affinity occurs in channel 1, while the absence of GP120 protein results in the majority of phages being recovered in channel 6. This system presents a precise and reproducible approach for the isolation and recovery of phages. In fact, in comparison to biopanning, which constitutes an affinity selection technique utilized to identify phage display variants possessing the desired binding characteristics towards a specific target [60], this method is characterized by its rapid execution, cost-effective, minimal reagent consumption, and reduced sample size.

#### 2.2.5. Cancer Stem Cell (CSC) Isolation and Culture

In this context, FMold was used to create a microfluidic device designed to isolate and cultivate tumor spheres as it more accurately replicates the physiological responses of CSCs in comparison to the alternative in vitro models. Furthermore, this approach significantly reduces the consumption of reagents and the volume of samples, which is particularly noteworthy given that the culture of CSCs requires costly reagents and substantial sample volumes [61]. Additionally, it functioned as a platform to assess the therapeutic responses of CSCs. The proliferation of individual spheres and their stemness in response to chemotherapeutic treatment (CT), specifically with doxorubicin and cisplatin, was systematically evaluated in bladder cancer cell lines (MB49-I and J82). The microdevice comprises an input and an output interconnected by four channels containing eighteen depth wells arranged in series within each line. Cells were seeded utilizing a micropipette. The pressure differentials between the inlet and outlet were simulated, and based on the parameters of cell seeding, no significant discrepancies were detected in the distribution of cells. Consequently, this setup facilitated the tracking of single-sphere growth over the time-course, enabled the performance of immunofluorescence investigations, and enabled the acquisition of high-quality RNA for subsequent molecular analysis [45].

Recently, the research group led by Eijan has devised and rigorously validated a microfluidic platform specifically designed for the culture of cancer stem cells (CSCs), which necessitates minimal volumes of both samples and reagents. The architecture of the microfluidic device was created through FMold structure replicated to ERmold and subsequently transferred to a PDMS replica. This innovative platform is compatible with immunofluorescence techniques and RNA extraction protocols, rendering it appropriate for both morphological and molecular analyses. The authors have illustrated that both murine and human breast cancer cell lines (LM38-LP and BRP6) were able to successfully generate spheroids within the microfluidic device and that treatment with doxorubicin or paclitaxel resulted in a significant diminution of spheroid size and proliferation rate, thereby indicating the effective targeting of proliferative cell populations. Furthermore, the researchers validated the capacity of the microfluidic device to sustain primary tumor cultures derived from LM38-LP tumors in BALB/c mice and subsequently extended this methodology to the realm of veterinary oncology by investigating three canine tumors. The capability to culture spheroids from veterinary tumors not only substantiates the platform’s cross-species applicability but also paves the way for advancements in comparative oncology, a discipline that integrates human and veterinary cancer research in order to enhance translational outcomes [62].

#### 2.2.6. Screening Drugs Against *Trypanosoma cruzi*

Another biomedical application of the FMold is the production of a concentration gradient generator microfluidic device that provides the tool to assess different drugs against *Trypanosoma cruzi* [44]. The device was placed under the microscope to acquire images. This platform enabled the simultaneous testing of six compound concentrations. Its automatic, high-precision image processing quickly and easily yielded extensive statistical data, streamlining the screening of new drugs.

**Figure 14:**
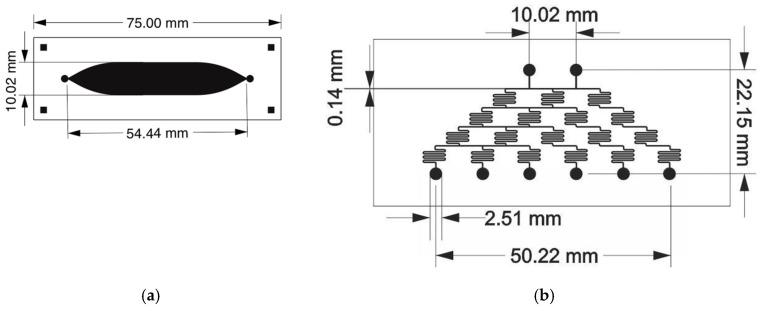
(**a**) The image displays two LOC devices: (**a**) the Cisternae device, with an internal volume of 1000 µL and its corresponding dimensions. (**b**) The Diluter LOC device, also shown with its dimensions, features two top inlets (one for reagent, one for solvent) and six bottom outlets. These outlets produce serial dilutions from right to left: 100%, 50%, 25%, 12.5%, 6.25%, and 0% [44]. License number: 5893810057664. License date: 21 October 2024. Licensed content publisher: Elsevier.

For this study, we designed and employed two different Lab-on-Chip (LOC) devices. One of these, known as “cisternae,” incorporated a sizable central cistern measuring 54.44 mm × 10.02 mm × 1 mm height, providing a 1000 mL volume (Figure 14a). The second LOC device, named “diluter” (Figure 14b), featured an integrated serial dilution generator (SDG). This SDG created six linear, two-fold dilutions (from 0% to 100%) from an initial concentrated solution. Its design relied on a resistive flow model with symmetric, thin microfluidic channels, enabling the diffusive mixing of soluble reagents.

To aid in assessing the trypanocidal impact of new drug candidates, the TR_KF program was developed to count moving parasites. The program’s GUI offers a visual breakdown of features detected during the tracking of image sequences. By classifying these features according to their maximum anticipated inter-frame distance, it enables an effective analysis of their unique motion patterns.

The diluter LOC device was employed to assess the trypanocidal activity of 18 drug candidates against intracellular amastigotes, following the same protocol established for BZN. Out of these, just three candidates demonstrated effectiveness: 2273-4 (IC_50_ 13.2 μM), 2160-X (IC_50_ 8.1 μM), and 2161-X (IC_50_ 39.3 mM).

After validating the LOC diluter device, researchers modified the experimental protocol from the cisternae LOC system to investigate Benznidazole’s (BZN) effects on intracellular *T. cruzi* amastigotes. This adaptation enabled the simultaneous testing of multiple drug concentrations within the diluter LOC. Vero cells were infected with *T. cruzi* trypomastigotes at an MOI of 5. Then, 1 × 10^5^ cells·well^−1^ of these infected cells per well were seeded into poly-D-lysine-treated LOC devices and given time to adhere.

**Figure 15:**
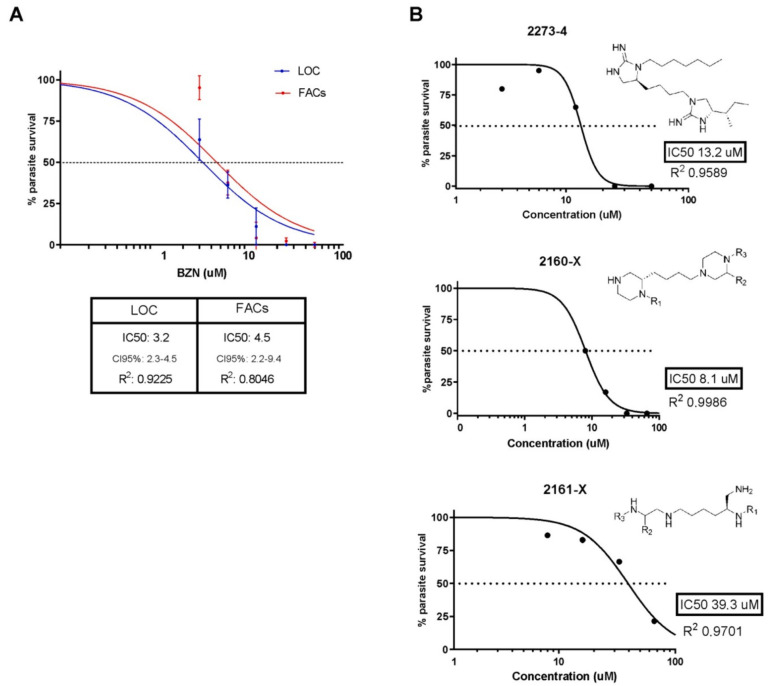
(**A**) Dose–response curves illustrating BZN’s inhibitory activity against *T. cruzi* amastigotes are generated using diluter LOC devices (blue circles) and flow cytometry (red circles). This graph represents data from eight independent experiments. An extra sum-of-squares F-test, comparing two nested models via least-squares regression, showed no significant difference between the curves (*p* = 0.2661, F = 1.28). CI95% refers to the 95% confidence interval. (**B**) Additional dose–response curves are generated with the diluter LOC device for candidate drugs exhibiting inhibitory activity against *T. cruzi* amastigotes. These graphs represent the combined results from three independent experiments for each compound. Used under the terms of the Creative Commons Attribution 4.0 license.

Following a twenty-four-hour incubation period, BZN (at a starting concentration of 50 μM) was introduced into the designated well of the LOC device to establish a concentration gradient. The experiment was monitored daily via video recordings for a duration of 72 h after drug application. Four videos were captured from randomly selected locations within each well, and parasite counts were determined with the program (TR_KF). Parasite survival percentages were subsequently calculated, which also allowed for the construction of dose–response curves. To further validate this innovative drug screening platform, the LOC device’s determination of BZN’s trypanocidal activity against *T. cruzi* amastigotes was directly compared against results derived from the established gold standard method of flow cytometry with GFP-expressing parasites. The determination of the half-maximal inhibitory concentration (IC_50_) was achieved through non-linear regression analysis using the “log(inhibitor) vs. normalized response”. The resulting IC_50_ values, along with their 95% confidence intervals (CI95), are displayed in Figure 15A. Furthermore, the dose–response curves were subjected to comparative analysis to calculate the F-value, a statistical measure of curve similarity. The dose–response curves derived from the new Lab-on-a-Chip (LOC) platform and the established flow cytometry technique displayed similar trends, with their respective IC50 values falling within a comparable range. Specifically, the LOC platform yielded an IC_50_ of 3.2 μM (CI95% 2.3–4.5) for Benznidazole (BZN), while the flow cytometry method resulted in an IC_50_ of 4.5 μM (CI95% 2.2–9.4) for the same drug (Figure 15A). To rigorously evaluate the comparability of these two methods, an extra sum-of-squares F-test was employed. This statistical test assesses whether a single curve can adequately fit all datasets. An F-value of 1.28, coupled with a *p*-value of 0.2661, indicates that there is no statistically significant difference between the curves generated by the two methods, thus demonstrating their comparability. This finding underscores the accuracy and reliability of the results obtained using the innovative microfluidic LOC platform, supporting its application as a viable tool for drug screening. Subsequently, to assess the trypanocidal activity of 18 potential drug candidates against intracellular *T. cruzi* amastigotes, the diluter Lab-on-a-Chip (LOC) device was employed, utilizing the established experimental protocol for Benznidazole (BZN). Out of the 18 compounds tested, only three demonstrated significant trypanocidal activity: compound 2273-4 with an IC_50_ of 13.2 μM, compound 2160-X with an IC_50_ of 8.1 μM, and compound 2161-X exhibiting an IC_50_ of 39.3 μM (Figure 15B).

#### 2.2.7. Droplet Storage Chip Fabrication for Gene Editing

FMold was utilized to fabricate a storage chip for droplets generated to improve clonal selection using hydrogels with a new method for gene editing using CRISPR-Cas9. The authors achieved successful genetic editing by isolating single cells in droplets and selecting them using extracellular matrix hydrogels in hiPSCs. This method is more efficient and versatile compared to other similar methods, and it can be adapted to different cell lines and work systems. The use of droplets and hydrogels results in high yields in isolating single cells, and the hydrogels are also beneficial for the long-term culture of single cells [46]. This study provides a more efficient clone selection method in terms of time. Furthermore, this approach avoids selection based on antibiotics.

#### 2.2.8. Fast and Optical Cell Detection Through a Hybrid Microchannel-Solid Micropore Device

**Figure 16:**
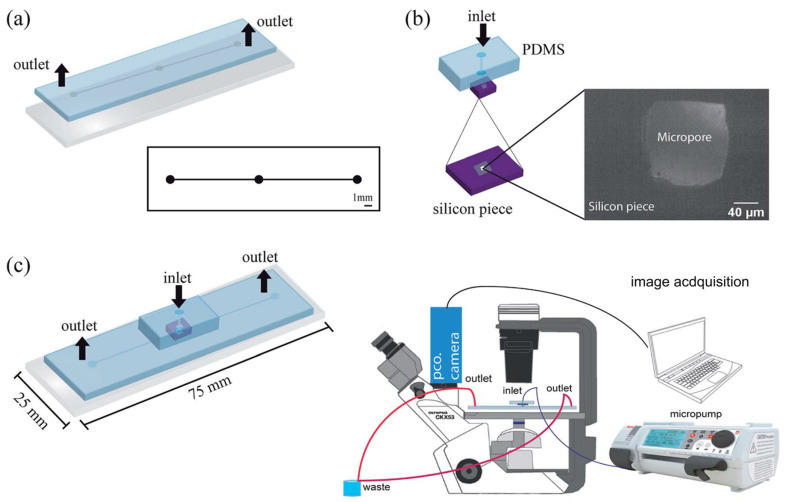
Setup fabrication for cellular enumeration: (**a**) the design of microchannels and the PDMS replica and a glass slide bonding, (**b**) the bonding and alignment of a micropore with a PDMS block measuring 5 mm in thickness, accompanied by a silicon piece exhibiting specified micropore dimensions located at the lower left, (**c**) a schematic representation of the microfluidic device and an assemble montage are provided [48]. Used under the terms of the Creative Commons Attribution-NonCommercial 3.0 Unported License.

**Figure 17:**
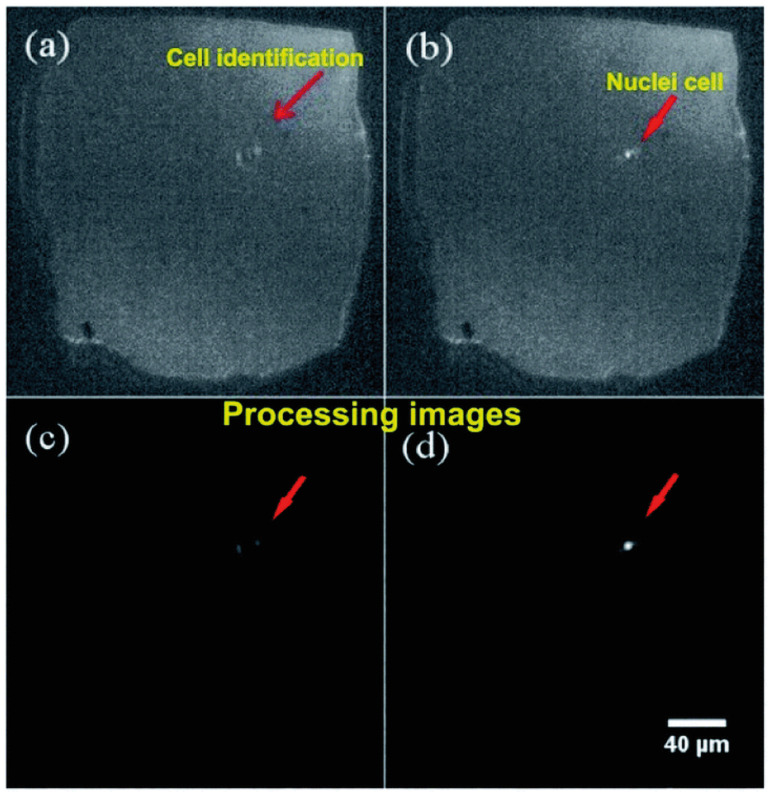
Identification of the entire cellular structure utilizing a bright field microscope: (**a**) image depicting the cellular structure, (**b**) image illustrating the nuclei within the cell, (**c**) image processing of the cellular structure conducted via ImageJ-FIJI software, (**d**) image processing of nuclei conducted via ImageJ-FIJI software. Micropore dimensions: approximately 150 μm × 150 μm [48]. Arrows indicate a cell passing through the micropore. Used under the terms of the Creative Commons Attribution-NonCommercial 3.0 Unported License.

FMold was employed to fabricate a PDMS microchannel with a thickness of 144 μm and a width of 500 μm (Figure 16a), thereby facilitating the development of a multilayer microfluidic cell counter equipped with a solid-state micropore produced via electrochemical etching [48]. Various sizes of micropores were achieved through chemical etching utilizing a 50 wt% KOH etchant at temperatures of 40, 60, and 80 °C, as well as voltages of 100, 500, and 1000 mV. Optical detection was positioned at the interface between the micropore and the PDMS transition zone. Figure 17 presents representative optical microscopy images of a single cell traversing the micropore. As cells traversed the micropore, images were captured and subsequently analyzed using ImageJ 1.54f software for the detection of cell nuclei.

Parameters such as pressure drop, shear stress, fluid viscosities and flow rates were incorporated into computational simulations to ascertain the optimal conditions (flow rate of 1.6 mL·min^−1^ and viscosity of 0.89 cP) for cell detection, thereby minimizing the risk of cell deformation or damage while obviating the requirement of a clean room-dependent lithography facility for microchannel fabrication. Moreover, expensive equipment, such as focused ion beam systems for the creation of the solid micropore, was not necessary. This integration was validated as a cost-effective methodology for cell detection and enumeration, thereby enabling any laboratory possessing fundamental chemical capabilities to conduct these experiments.

#### 2.2.9. Development of an Algorithm to Quantify, Count and Measure Spheres Derived from Cancer Stem Cells Automatically

**Figure 18:**
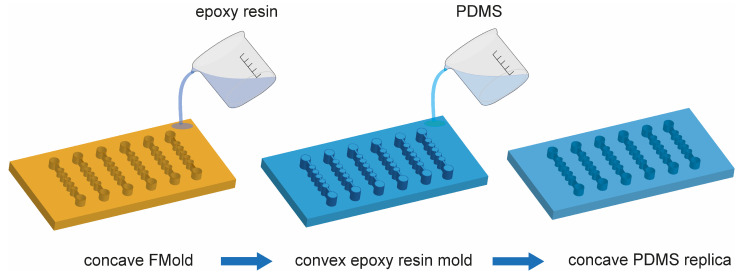
PDMS replica fabrication for sphere derived from CSC culture.

**Figure 19:**
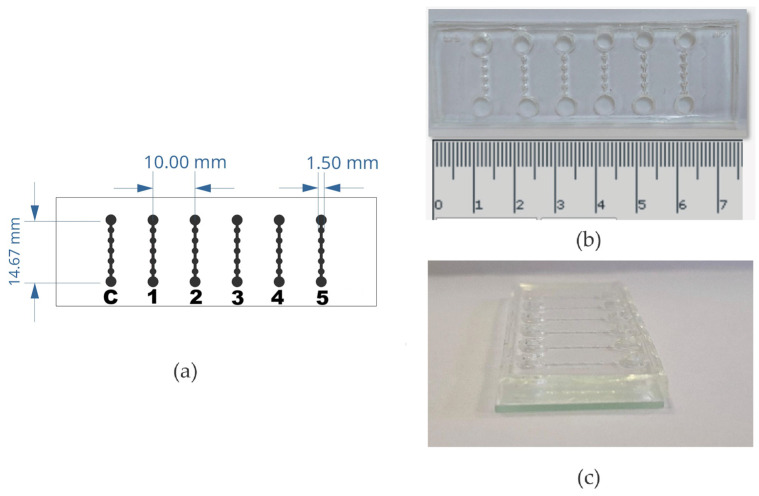
Microfluidic device architecture: (**a**) The design of the microfluidic device consists of 6 channels. Each individual channel is formed by an inlet, 5 chambers, and an outlet. (**b**) Topographical representation of the microfluidic device with measurement scale in centimeters. (**c**) Lateral perspective of the microfluidic device [47]. Used under the terms of the Creative Commons Attribution License 4.0 license.

**Figure 20:**
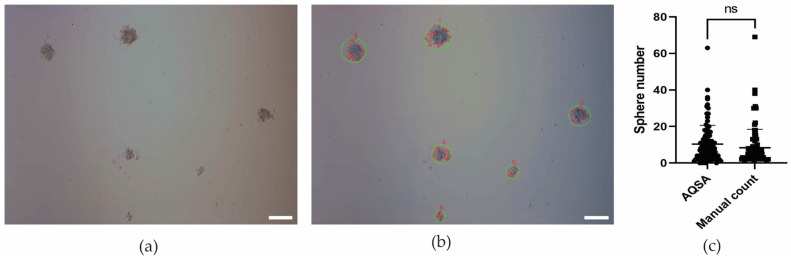
Detection, identification, and quantification of spheres within a 12-well plate: (**a**) Original image captured at 10× magnification. (**b**) Identified spheres are highlighted in green and enumerated in red. U251 human glioblastoma cell line utilized. Scale bar measures 200 μm. (**c**) Comparative analysis of sphere counts between AQSA and manual enumeration conducted on one hundred 10× images of the U251 human glioblastoma cell line. Statistical evaluation via Student’s *t*-test (*p* = 0.167); ns denotes the absence of a statistically significant difference (*p* > 0.05) [47]. Used under the terms of the Creative Commons Attribution License 4.0 license.

Concave FMold was applied to fabricate a convex epoxy resin mold to obtain a PDMS replica as illustrated in Figure 18 to produce the microfluidic device (Figure 19) that enabled the isolation and culture of spheres derived from cancer stem cells dissociated from veterinary patient tumors. The microfluidic chip comprises six channels, enabling the performance of control and the testing of different cell seeding concentrations to form spheres in the same device. In addition, the size of the microchambers fits the 10× objective field of view. Then, entire microchamber images can be acquired. This is really useful for sphere tracking in the time course. These spheres were detected, quantified, and measured by the Automatic Quantification of Spheres Algorithm (AQSA), which is a computer program that allows to automatically find, count, and measure the area of spheres in images (Figure 20). It provides a low computational cost and accessible tool for labs in lower-income countries to analyze images of spheres reliably. Furthermore, it has a user-friendly interface where researchers can input details about the images, like the magnification level and file format. This information helps the program accurately measure the size of the spheres [47].

## 3. Other Applications of Flexography in Science

Rahul et al. 2023 [63] showed how to build microdevices with a photopolymer sheet used to create microchannels by exposing them to UV light through a mask and then etching away the unexposed areas. They placed another sheet of the same material on top and bonded it using pressure and UV light. This approach was successfully evaluated for cell culture, biomolecule visualization, and droplet mixing dynamics.

Digital flexography, with its ability to print precise patterns on flexible substrates, has become a promising tool for the fabrication of microfluidic devices [64]. This technology allows for the creation of channels, chambers, and other microfluidic components directly onto substrates such as polymer films, paper, and even textiles.

### 3.1. Diagnostic Devices

Digital flexography has also been used to fabricate microfluidic diagnostic devices for a variety of diseases. These devices can be used in low-resource settings, where access to conventional laboratories is limited. This aspect is deeply reviewed by Davis et al. 2021 [65]. Furthermore, to make good-quality healthcare available and affordable for everyone in both higher-income and lower-income countries, diagnostic tests that are both accurate and inexpensive are needed [66].

### 3.2. Chemical Analysis

Flexographic printing technology has been evaluated to produce fluidic structures in paper [67], continuous conductive grids [68], polymer solar cell modules [69], and for multi-busbar solar cells [70]. This research group determined that flexographic printing is a cost-effective way to apply the front-side metallization to solar cells. It is faster than traditional screen printing and uses less of the expensive silver ink. In addition, the fine lines that it creates reduce shading and improve efficiency. Tests showed that flexography can create the very thin lines needed for solar cell contacts. Lines as small as 33 μm were successfully printed using this technique by introducing an intermediate drying step between two printing passes [70].

FLEXEL NX has also proven to be an effective substrate for the fabrication of polymer optical waveguides, serving as fundamental components within planar optronic systems, which represent optical technologies designed for integrated, locally resolved sensing functionalities. The high-throughput capabilities of flexography facilitated the productive mass manufacturing of the aforementioned components for tailored sensor systems [71]. These researchers ascertained the minimum feature size achievable during the engraving process of the printing plate. In the context of photopolymers that exhibit no oxygen inhibition such as Kodak, FLEXEL NX is capable of attaining a minimum width of 10 μm. The upper threshold for line width in the process is constrained by the phenomenon of viscous fingering [72], which manifests when the line width exceeds 900 μm. The occurrence of viscous fingering results in an amplification of surface undulations. Nevertheless, this phenomenon can yield positive outcomes, as described in the next application.

### 3.3. Biofabrication

**Figure 21:**
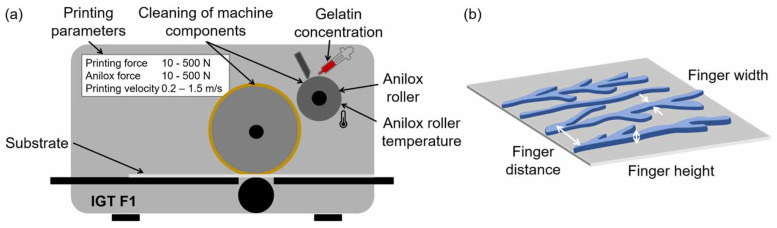
Schematic representation of the constituents of the printing machine, alongside the printing parameters (**a**) that impact the morphology of the resultant finger-like formations (**b**). Image reproduced from Brumm et al. 2022 [73] used under the terms of the Creative Commons Attribution 4.0 license.

Flexographic printing technology has been used to efficiently create patterns on substrates encompassing several square centimeters of area utilizing a sacrificial bioink that replicates the structural characteristics of natural vascular networks in both morphology and dimensions [73]. These authors capitalized on the phenomenon of spontaneous viscous fingering pattern formation to fabricate networks that closely resemble vascular networks within a sacrificial gelatin medium (Figure 21).

## 4. Current Challenges and Limitations

Despite the significant advantages offered by FMold and ERmold systems for mold replication and microfabrication, several challenges and limitations persist that can affect the reproducibility, resolution, and scalability of microstructures.

### 4.1. Material Compatibility

FMold demonstrates compatibility with a broad range of solvents and ink types—including water-based, solvent-based, UV-cured, and EB-cured inks [74]. However, UV-curable flexographic inks are destructive to the printing plate’s surface. In addition, solvents such as ethyl acetate induce the swelling of the photopolymer substrate and may even lead to the partial dissolution of the photopolymer material [50]. Concerning its intermediate epoxy counterpart (ERmold), it is recognized for robust chemical resistance [51]. However, ERmold’s compatibility with biological samples is limited as it does not allow gas exchange (CO_2_). However, epoxy resin microfluidic devices have been fabricated for liquid chromatography, anaerobic bacterial culture, and polymerase chain reaction applications [49].

### 4.2. Aspect Ratio and Feature Resolution Constraints

The achievable aspect ratio and resolution of features differ depending on the FMold system used. FLEXCEL SRH allows for high-aspect ratios up to 60, enabling structures as tall as 1500 μm with minimum feature widths of 25 μm; however, features below 25 μm could not be reliably fabricated [25]. In contrast, FLEXCEL NX yields a lower aspect ratio of 13, supporting features up to 274 μm in height and 21 μm in width [28]. These differences limit design flexibility and pose constraints on fabricating very fine or deep structures, depending on the system.

### 4.3. Durability and Replication Fidelity

Although epoxy sub-masters (ERmolds) allow for at least 10 high-fidelity PDMS replicas—verified through profilometry and SEM imaging—the FMold itself has only been characterized for up to three ERmold replications [27]. While more may be possible, the practical upper limit of FMold durability remains undefined, as it is no longer needed after the ERmold is produced. Therefore, the understanding of FMold’s lifespan and reusability in extended manufacturing cycles is limited. However, Tomašegović 2016 [50] reports in her doctoral thesis that most flexo-printing plates are designed to produce up to a million copies before they start showing signs of mechanical wear.

### 4.4. Challenges in Reproducible Multi-Level Structure Fabrication

The generation of multi-level microstructures relies heavily on understanding and controlling the photopolymer’s behavior under UVA exposure. Studies have shown an inverse correlation between UVA exposure time and structure height/depth for both positive and negative features. Yet, consistent depth formation becomes problematic when line widths exceed 400 μm due to the emergence of trapezoidal geometries and overlapping sidewalls [28]. These effects introduce variability and complicate precise control over multi-level features, especially in complex designs.

### 4.5. Potential Oxygen Inhibition in Alternative Photopolymer Systems

One of the key advantages of the FLEXCEL NX system lies in its oxygen inhibition barrier, achieved using a thermal imaging layer (TIL) whose lamination constitutes the method employed to eradicate oxygen during the ultraviolet exposure phase. This barrier enables the creation of flat top dots and ensures precise 1:1 image transfer during photopolymerization [75]. However, this benefit may not extend to other photopolymer systems lacking a similar barrier mechanism. Without such a feature, oxygen inhibition could compromise the resolution and fidelity of patterned features, particularly in air-exposed environments, limiting the versatility of mold fabrication methods beyond the FLEXCEL NX platform.

## 5. Conclusions

Current research on FMold for microfluidics is focused on enhancing the resolution and precision of the printing process to fabricate even smaller and more complex microfluidic structures. This includes exploring new materials and techniques to improve the printing resolution beyond the current limitations. Perez’s research group is working on strategies to improve resolution. Currently, 4 μm resolution has been achieved.

Automation and integration are also key focuses for future developments in flexographic printing for microfluidics. Automating the fabrication process can improve the reproducibility and throughput of microfluidic device production. Moreover, integration with electronic components can lead to the development of smart microfluidic systems with enhanced capabilities, such as the real-time monitoring and control of fluid flow.

The future of flexographic printing in microfluidics is promising. By tackling existing challenges and pursuing new research avenues, the technology is set to revolutionize the field. This will lead to the creation of innovative and accessible microfluidic devices with a broad range of applications.

FMold has emerged as a versatile and promising technique for microfluidic device fabrication, offering several advantages such as global availability, cost-effectiveness, scalability, and rapid prototyping. These advantages reduce inequalities through low-cost and accessible technology, which aligns with sustainable development goal 10.

This review has explored the fundamentals of flexographic printing, its adaptation for microfluidics, and its diverse applications in areas ranging from chemistry to biomedicine.

The ability of flexographic printing to create microchannels with high resolution and precision has enabled the development of innovative microfluidic devices for a wide range of applications, from diagnostic devices for low-resource settings to microfluidic platforms for cancer stem cell research and drug screening, which align with sustainable development goal 3. Overall, flexographic printing is making significant contributions to the advancement of microfluidics.

The integration of nanoparticles, the development of functional inks holds tremendous potential for the future of flexographic printing in microfluidics. These advancements are expected to lead to the creation of more sophisticated and versatile microfluidic devices with enhanced functionalities and broader applications.

In conclusion, flexographic printing is revolutionizing the fabrication of microfluidic devices, enabling laboratories that do not have the microfabrication facilities, such as biology laboratories or research laboratories in lower-income countries, to be able to generate fast and locally their own microfluidic devices at low cost. As research progresses and technology evolves, flexographic printing is poised to play an even greater role in the development of innovative microfluidic solutions for a wide range of applications, from diagnostics and chemical analysis to biomedicine and beyond.

## Figures and Tables

**Table 1:** FLEXCEL NX plate layer function.

	Description	References
Base Film (PET)	Provides support and dimensional stability to the photopolymer layer of the FLEXCEL NX plate. This is part of the final plate structure.	[1]
Laminated Interface	The critical interface created when the TIL is laminated onto the FLEXCEL NX plate before UV exposure. This eliminates oxygen inhibition.	[7,8]
Thermal Imaging Layer (TIL)	A separate film with a special coating that is thermally imaged to create a high-resolution mask.	[9]
FLEXCEL NX Plate	The main photopolymer material that is selectively hardened by UV light to form the relief image. This becomes the final printing plate.	[9]

**Table 2:** Comparison of mold fabrication technologies based on (Scott and Ali 2021 [26]).

Technology	Minimum Feature Size (µm)	Resolution	Typical Aspect Ratio	Achievable Roughness Ra (nm)	1 cm^2^ Price (USD)	References
FMold from FLEXCEL SRH	25	10 µm	60	23	0.03	[25]
FMold from FLEXCEL NX positive relief (convex)	357.6	10 µm	13	7.4	0.07	[27,28]
FMold from FLEXCEL NX negative relief (concave)	114	10 µm	13	29.8	0.07	[27,28]
Laser ablation	<1	~1	<10	100	0.02	[29,30]
Focus ion beam	40 nm	5 nm	10	0.58	>65	[31,32]
MEMS process	Some µm	NA	<40	10	>100	[33]

**Table 3:** Advantages and disadvantages of FMold and ERmold.

	FMold	ERmold
Advantages		
Short-time mold fabrication [27]	X	X
Clean room-free [25]	X	X
Large substrate (1270 2062 mm^2^) [28]	X	
Heights up 1500 µm [28]	X	X
A diverse array of molds exhibiting a broad spectrum of dimensions [28]	X	
High-throughput replication [27]	X	X
Low price [25]	X	X
Worldwide accessible [25,27,28]	X	X
Monolithic mold that is not prone to delamination [27]	X	X
Durable [27,49,50]	X	X
Reusable [25,27]	X	X
Chemical resistance [51]		X
Disadvantages		
Resolution (10 µm) [28]	X	X
Widths less than 20 µm are not resolved yet [8]	X	X
Sidewall inclination [25,27,28]	X	X
Requires plasma-enhanced chemical vapor deposition for SiO_2_ coating [25,27]	X	

## Data Availability

Not applicable.

## Supplementary Materials

The following supporting information can be downloaded at https://www.mdpi.com/article/10.3390/polym17131723/s1, Table S1: FLEXCEL companies around the world.

## Abbreviations

The following abbreviations are used in this manuscript:

AQSA	Automatic quantification of spheres algorithm
BZN	Benznidazole
Ca	Capillary number
CNC	Computer numerical control
CI	Confidence intervals
CSCs	Cancer stem cells
CT	Chemotherapeutic treatment
DTIR	A high-quality digital no-process film imaged with KODAK SQUARESPOT Imaging Technology
EBOV	Ebola virus
EVD	Ebola virus disease
ERmold	Epoxi-resin mold
EOR	Enhanced oil recovery
FMold	Flexographic printing plate mold
FMold-T	SiO_2_-coated mold
GP	Glycoprotein
GUI	Graphical user interface
hiPSC	Human-induced pluripotent stem cell
HIV	Human immunodeficiency virus
HMDS	Hexamethyldisilazane
LAMS	Laser ablation mask systems
LED	Light-emitting diode
LM bioreactor	Large-area microfluidic bioreactor
LOC	Lab-on-a-chip
mAb	Monoclonal antibody
MOI	Multiplicity of infection
PDMS	Polydimethylsiloxane
PET	Polyethylene terephthalate
PEVCD	Plasma-enhanced chemical vapor deposition
PV	Poral volume
SDG	Serial dilution generator
SEM	Scanning electron microscopy
TIL	Thermic imaging layer
UV	Ultraviolet
