# Supplementary material for: Photopolymer Flexographic Printing Plate Mold for PDMS Microfluidic Manufacture
Source: Polymers (Basel). 2025 Jun 20;17(13):1723. doi: 10.3390/polym17131723 (PMC12251789; doi:10.3390/polym17131723)
Supplement: Supplementary file 1 [file polymers-17-01723-s001.zip › polymers-3654812-supplementary.pdf]

Supplementary information

Table S1.

Company name	Address	Country	Web site
Imagex	Parque Leoncio Prado 144, Magdalena del Mar, Lima - Perú	Peru	https://www.imagex.com.pe/flexo
Proveedora flexografica	Dr. Jesús Ma. González No.220 Col. San Jerónimo Monterrey, N.L. 64640	Mexico	https://flexografica.com/contacto/
Sumiflex	Calle París, Torre Profesional La California Planta Baja, La California Norte, Caracas	Venezuela	https://www.sumiflex.com/
XSYS Germany GmbH	Industriestraße 1, 77731 Willstätt, Germany	Germany	https://xsysglobal.com/?utm_medium=cpc&utm_source=google&utm_campaign=Brand&utm_term=xsys&gad_source=1&gclid=CjwKCAiAgoq7BhBxEiwAVcW0LBleL6t6bmf5TXJhZ61UvwFjX8ME4RLA_edvRga47vjLv7pAX9XEdRoCYpgQAvD_BwE
FLEXOCODINTESA	Guayaquil Km. 7 1/2 vía a Daule, sector La Prosperina Solar 9 Mz. 8 Guayaquil, Ecuador	Ecuador	https://www.flexocodintesa.com/impresion.php
El empaque	Cll 73 # 10-83 Torre C Piso 4 Bogotá, Colombia	Colombia	https://www.elempaque.com/es/productos/impresion-flexografica
Ferrostaal Graphics SPA	Calle Nueva 1899, Huechuraba, Santiago - Chile.	Chile	https://www.ferrostaal-graphics.com/producto/kodak-flexcel-nx/
U. GÜNTHER GMBH	LENTFÖHRDENER WEG 21 22523 HAMBURG	Germany	https://www.guenther-prepress.com/
FLEXOO GmbH	Speyerer Straße 4 69115 Heidelberg	Germany	https://www.flexoo.de/

	Germany		
Monarch Color Corp	5327 Brookshire Blvd, Charlotte, NC 28216, United States	United States	https://www.monarchcolor.com/?gad_source=1&gclid=CjwKCAiAgoq7BhBxEiwAVcW0LHxv8MvXgVawtKhWLzv1uwm6wN82DSE90Q8bVR9Wv_2Clm-4u-3uARoCJ74QAvD_BwE
Splash Graphics, Inc	7001 S Adams St Willowbrook, IL 60527	United States	https://splash-graphics.com/flexography/
Pure Infinity	Headquarters<br>Lhee 15a<br>7991 PE<br>Dwingeloo	Nederlands	https://www.pureinfinity.nl/de/flexographic-printing-company-belgium/
Flexo24	Via Giorgione, 8 – 31056 Roncade (TV) –	Italy	https://www.flexo24.com/international/
Tait Graphics	The Studio 11 High Street Dry Drayton Cambridge CB23 8BS	United Kingdom	http://www.tait-designs.com/
Alphaprint	Av. Ceci, 2193 - Planalto Paulista, São Paulo - SP, 04065-004	Brasil	https://alfaprint.com.br/
Reproflex3	Cramlington<br>27 Moorland Way<br>Cramlington<br>NE23 1WE	United Kingdom	https://reproflex3.com/
Flint CPS Inks Germany GmbH	Gaugrafenstrasse 11-13, 60489 Frankfurt, Germany	Germany	https://www.flintgrp.com/contact/locations/emea/germany/
Lucky Huaguang Graphics	718 Chezhan South Road Nanyang, Henan 473051 China	China	https://www.hgfilm.com.cn/en/product.html?cate=327&level=774
TechNova Imaging Systems	Laxmi Mills Estate, Off Dr. E. Moses Road, Mahalaxmi, Mumbai 400 011, Maharashtra, India	India	https://www.technovaworld.com/

Kodak	4-10-13 Higashishinagawa, Shinagawa-ku Tokio 140-0002 Japón	Japan	https://www.kodak.com/ja/
Suzhou AoLiDe Co., Ltd	NO.27, XingNan Road, WuZhong Economic Development Zone, Suzhou, Jiangsu, China	China	https://www.szaolide.com/analog-flexographic-printing-plate/1-14mm-analog-flexographic-flexo-photopolymer-plate.html?gad_source=1&gclid=CjwKCAiAgoq7BhBxEiwAVcW0LOOTKa3EJIaBHi-m7Dde1WiqBEtk39SHKMDjhpCulyRpDSDPnEdv7RoCntsQAvD_BwE
